# Brown Algae (Phaeophyceae) from the Coast of Madagascar: preliminary Bioactivity Studies and Isolation of Natural Products

**DOI:** 10.1007/s13659-015-0068-0

**Published:** 2015-09-10

**Authors:** Marie Pascaline Rahelivao, Margit Gruner, Hanta Andriamanantoanina, Ingmar Bauer, Hans-Joachim Knölker

**Affiliations:** Department Chemie, Technische Universität Dresden, Bergstraße 66, 01069 Dresden, Germany; Centre National de Recherche sur l’Environnement, MESupRes, BP 1739, 101 Antananarivo, Madagascar

**Keywords:** Brown algae, Steroids, Terpenoids, NMR spectroscopy, Antimicrobial activity

## Abstract

**Abstract:**

Eight species of brown algae (Phaeophyceae) from the coast of Madagascar have been investigated for their chemical constituents. Fucosterol (**3**) was obtained as the most abundant compound. The brown alga *Sargassum ilicifolium* was the source for the first isolation of the terpenoid C_27_-alcohol 1,1′,2-trinorsqualenol (**1**) from marine sources. From *S. incisifolium* we isolated the highly unsaturated glycolipid 1-*O*-palmitoyl-2-*O*-stearidonoyl-3-*O*-β-D-galactopyranosylglycerol (**4**) and we report the first full assignment of its ^1^H and ^13^C NMR data. Apo-9′-fucoxanthinone (**8**) along with 24-ketocholesterol (**5**), (22*E*)-3β-hydroxycholesta-5,22-dien-24-one (**6**), and saringosterol (**7**) were obtained from *Turbinaria ornata*. The crude extracts of all eight species of brown algae exhibited a pronounced antimicrobial activity against the Gram-positive bacteria *Bacillus cereus*, *Staphylococcus aureus*, and *Streptococcus pneumoniae*.

**Graphical Abstract:**

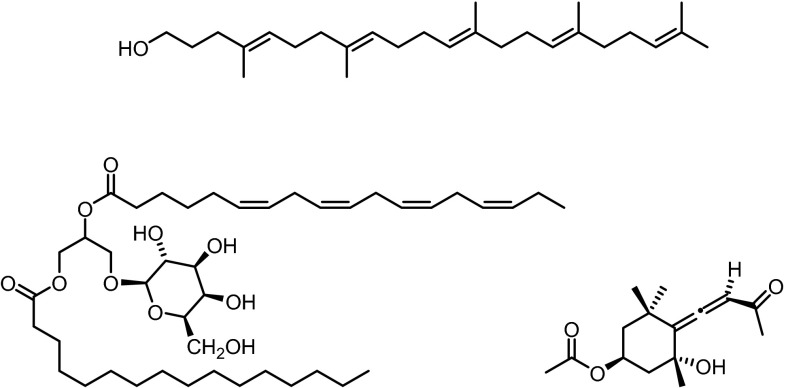

**Electronic supplementary material:**

The online version of this article (doi:10.1007/s13659-015-0068-0) contains supplementary material, which is available to authorized users.

## Introduction

Marine algae have come more and more into focus as promising sources of novel and potentially bioactive primary and secondary metabolites [1–8]. The coastal waters of Madagascar, the fourth largest island in the world with nearly 5000 km of coastline, are inhabited by a wealth of marine organisms. Recently, we published a study on the chemical composition of various red algae collected at diverse places at the coast of Madagascar [9]. Among others, brominated indols, A-ring contracted steroids and debilone have been isolated from these species. To date, only few representatives of brown algae from Madagascar have been investigated for their chemical constituents. For the brown algae *Spatoglossum* sp., *Sargassum* sp. 1, *Sargassum* sp. 4, *Zonaria* sp., *Chnoospora* sp., and *Spatoglossum* sp. Andriamanantoanina and Rinaudo identified alginate polymers which are composed of (1 → 4)-β-d-mannuronic acid (M) and (1 → 4)-α-l-guluronic acid (G) units [10, 11]. They examined the influence of the block distribution (MM and GG) along the alginate chain on their gel forming ability under acidic conditions. Recently, Andriamanantoanina and coworkers investigated the polysaccharide fraction of *Sargassum* sp., *Turbinaria* sp., and *Hormophysa* sp. and identified gel forming alginates [12]. The present study aims at the investigation of the chemical constituents of the nonpolar fractions from brown algae collected at the coast of Madagascar.

## Results and Discussion

### *Sargassum ilicifolium*

**Fig. 1:**
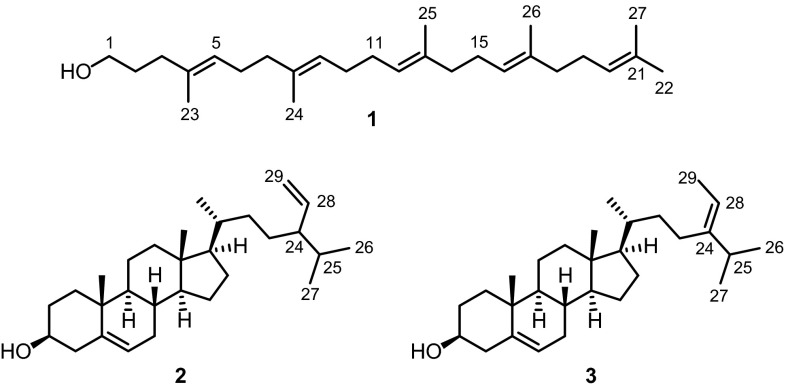
Structures of 1,1′,2-trinorsqualenol (**1**), stigmasta-5,28-dien-3β-ol (**2**) and fucosterol (**3**)

Samples of brown seaweeds of *Sargassum ilicifolium* were extracted with methanol. Bioassay-guided fractionation of the crude methanol extract of *S. ilicifolium* led to the isolation of 1,1′,2-trinorsqualenol (**1**) along with stigmasta-5,28-dien-3β-ol (**2**) and fucosterol (**3**) (Fig. 1). The latter has long been known as the predominant sterol of Phaeophyceae [13, 14]. Compound **1** was isolated as colorless oil. The molecular mass of 386 was obtained from EI (*m*/*z* = 386 [M]^+^) and ESI mass spectra (*m*/*z* = 404.4 [M + NH_4_]^+^). This information combined with ^1^H and ^13^C NMR data supported the molecular formula C_27_H_46_O. The ^1^H NMR spectrum displayed signals for 46 protons including five vinylic protons at *δ* = 5.09–5.15 ppm (m, 5 H, C=CH).

The ^13^C NMR and DEPT data of **1** revealed the presence of six methyl groups (C-22, C-23, C-24, C-25, C-26, C-27), five quaternary olefinic C-atoms (C-4, C-8, C-13, C-17, C-21), five olefinic CH moieties (C-5, C-9, C-12, C-16, C-20), one HO–CH_2_ group (C-1), and ten CH_2_ groups (C-2, C-3, C-6, C-7, C-10, C-11, C-14, C-15, C-18, C-19). A proton to carbon assignment could be achieved by an HSQC experiment. The location of the methyl groups and the olefinic moieties could be assigned on the basis of COSY, HMBC, and NOESY correlations. The *E* configuration of the double bonds in positions 4, 8, 12, and 16 was deduced from NOESY experiments by correlation of the methyl groups (H_3_-23, H_3_-24, H_3_-25, H_3_-26) with the respective allylic protons and absence of a correlation with the vinylic protons. The data obtained from 1D and 2D NMR experiments unambiguously led to the elucidation of compound **1** as (4*E*,8*E*,12*E*,16*E*)-4,8,13,17,21-pentamethyldocosa-4,8,12,16,20-pentaen-1-ol, which is also known as 1,1′,2-trinorsqualenol, with full assignment of all ^1^H and ^13^C NMR signals. Our ^1^H and ^13^C NMR data are in good agreement with those of the synthetic product described by Gref and coworkers [15] whereas the original data of Prestwich et al. [16] deviate slightly (Tables 1, 2).

**Table 1:** ^1^H NMR spectroscopic data of **1**

Position	From *Sargassum ilicifolium* *δ*_H_ (600 MHz, CDCl_3_)	Synthetic product [15] *δ*_H_ (400 MHz, CDCl_3_)^a^	Synthetic product [16] *δ*_H_ (300 MHz, CDCl_3_)^a^
5	5.15 (m, 1 H)^b^	5.03–5.23 (m, 5 H, C=CH)	5.06 (br m, 5 H, C=CH)
9	5.13 (m, 1 H)^b^		
12, 16	5.11 (m, 2 H)^b^		
20	5.09 (m, 1 H)^b^		
1	3.62 (t, *J* = 6.6 Hz, 2 H)	3.63 (t, *J* = 6.4 Hz, 2 H, OCH_2_)	3.48 (t, *J* = 6.5 Hz, 2 H, OCH_2_)
6	2.07 (m, 2 H)^b^	1.90–2.14 (m, 18 H, 9 CH_2_)	1.92 (br m, 18 H, C=CCH_2_)
19	2.06 (m, 2 H)^b^		
3, 14	2.05 (m, 4 H)^b^		
10, 11	2.01 (m, 4 H)^b^		
7	1.98 (m, 2 H)^b^		
15, 18	1.97–1.95 (m, 4 H)^b^		
27	1.67 (s, 3 H)	1.68 (s, 3 H, CH_3_)	See positions 22, 24, 25, 26
2	1.66 (m, 2 H)^b^	1.52–1.73 (m, 17 H, 6 CH_3_, CH_2_) [15]	1.58 (br s, 6 H, C-2 CH_2_, C-22 CH_3_)
23	1.60 (m, 3 H)^b^		
22, 24, 25, 26	1.59 (s, 12 H)^b^		1.55, 1.51 (br s, 15 H, C-23, C-24, C-25, C-26, C-27 CH_3_)
OH			2.91 (br s, 1 H, OH)

^a^Assignment based on comparison with our data from *Sargassum* *ilicifolium*. Original assignment in parentheses

^b^Chemical shift determined from the HSQC spectrum

**Table 2:** ^13^C NMR spectroscopic data of **1**

Position	From *Sargassum ilicifolium* *δ*_C_ (150 MHz, CDCl_3_)	Synthetic product [15] *δ*_C_ (100 MHz, CDCl_3_)^a^	Synthetic product [16] *δ*_C_ (75 MHz, CDCl_3_)^a^
13	135.14	135.1 (C)	134.67
17	134.95	134.9 (2 C)	134.58 134.48
8	134.92		
4	134.58	134.5 (2 C)	134.19 (C-4, C-8, C-13, C-17)
21	131.26	131.2 (C)	130.70 (C-21)
5	124.84	124.8 (CH)	124.44
9	124.43	124.4 (2 CH)	124.27 124.22
20	124.39		
12, 16	124.26, 124.25	124.2 (2 CH)	124.12 (C-5, C-9, C-12, C-16, C-20)
1	62.84	62.8 (CH_2_, CH_2_O)	62.12 (C-1)
11	39.74	39.7 (CH_2_)	See position 10
15	39.72		See positions 6, 19
18			39.56
7	39.65	39.6 (CH_2_)	35.77
3	36.00	35.9 (CH_2_)	31.44
2	30.69	30.7 (CH_2_)	30.66 (C-2, C-3, C-7, C-14, C-18)
10	28.25	28.2 (2 CH_2_)	28.08 (C-10, C-11)
14	26.76	26.7 (CH_2_)	See positions 2, 3, 7, 18
19	26.65	26.6 (CH_2_)	26.60, 26.42, 25.43 (C-6, C-15, C-19)
6	26.55	26.5 (CH_2_)	
27	25.69	25.6 (2 CH_3_)	See positions 24, 25, 26
22	17.67	17.6 (CH_3_)	22.50 (C-22)
24	16.03	15.9 (2 CH_3_)	15.74, 15.61, 13.88
25, 26	15.99		(C-24, C-25, C-26, C-27)
23	15.84	15.8 (CH_3_)	17.38 (C-23)

^a^Assignment based on comparison with our data from *Sargassum* *ilicifolium*. Original assignment in parentheses

1,1′,2-Trinorsqualenol (**1**) was first obtained as ^3^H-labeled isotopomer by Corey et al. from 2,3:22,23-dioxidosqualene [17]. Prestwich and coworkers synthesized the parent compound from 2,3-oxidosqualene by periodic acid cleavage of the epoxide and subsequent reduction of the aldehyde with sodium borohydride [16]. 1,1′,2-Trinorsqualenol (**1**) was found to be an active squalene oxidase inhibitor with an IC_50_ value of 4 µM [16]. This finding induced several studies on the squalene oxidase inhibiting activity of 1,1′,2-trinorsqualenol (**1**) and congeners (e.g. Ref. [18, 19]). 1,1′,2-Trinorsqualenol (**1**) is readily available by synthesis from 2,3-oxidosqualene. However, there is only one previous report from 2010 by Li et al. who detected 1,1′,2-trinorsqualenol (**1**) as a natural product by GC–MS analysis of *Zanthoxylum* oil [20]. In the present work, we describe the first isolation of 1,1′,2-trinorsqualenol (**1**) from a marine organism and its full spectroscopic characterization.

Our sample of stigmasta-5,28-dien-3β-ol (**2**) from *S. ilicifolium* was obtained as an amorphous solid with a melting point of 119–120 °C. ^1^H and ^13^C NMR data are in agreement with those of the synthetic sample reported by Djerassi et al. [21] and slightly deviate from those of the product isolated from *Saxifragamontana* H [22]. The ^1^H NMR spectrum shows two sets of signals for H_3_-26, H_3_-27, and H-28 which indicates the presence of a mixture of C-24 epimers in a ratio of 1:1. This also accounts for the lower melting point of 119–120 °C of **2** obtained from *S.ilicifolium* compared to the pure epimers [(24*R*)-isomer: mp = 132–133 °C [21], (24*S*)-isomer: mp = 141–142 °C [21] ]. Stigmasta-5,28-dien-3β-ol (**2**) was first obtained by Ikekawa via reduction of the corresponding propargylic alcohol [23]. Djerassi and coworkers synthesized both C-24 epimers of stigmasta-5,28-dien-3β-ol (**2**) and proved that the sample of Ikekawa was a 1:1 mixture of both isomers [21]. Isolations from living organisms were reported from the marine sponge *Haliclona* sp. [24], from the perennial herbaceous plant *Saxifragamontana* H. [22] and others. In 2014, stigmasta-5,28-dien-3β-ol (**2**) was isolated from the Chinese brown alga *Sargassumthunbergii* by the group of Guo [25]. The present report describes the first isolation of stigmasta-5,28-dien-3β-ol (**2**) from the brown alga *S.ilicifolium*.

In an antimicrobial agar diffusion test, the crude extract of *S. ilicifolium* was found to be very active against the Gram-negative bacteria *Shigella boydii* and *Klebsiella oxytoca*, the Gram-positive bacteria *Streptococcus pneumoniae* and *Staphylococcus aureus*, and the yeasts *Candida membranaefaciens*, *Trichosporon mucoides*, and *Cryptococcus neoformans* (Table 3). Significant activity was also observed against the Gram-negative bacterium *Enterobacter cloacae* and the Gram-positive bacterium *Bacillus cereus*. No activity was found against the Gram-negative bacteria *Pseudomonas aeruginosa*, *Escherichia coli*, *Salmonella enteridis*, and the yeast *Candida albicans*.

**Table 3:** Antimicrobial activities of the crude extracts of brown algae by agar diffusion test^a^

	Zone of inhibition (Ø in mm)^b^							
Microbes	*Hormophysa cuneiformis*	*Sargassum ilicifolium*	*Sargassum incisifolium*	*Sargassum polycystum*	*Sargassum *sp.	*Turbinaria conoides*	*Turbinaria decurrens*	*Turbinaria ornata*
Gram-negative bacteria								
*Enterobacter cloacae* ATCC 700323	6.5	8	6	6.5	6.5	6.5	8	9
*Klebsiella oxytoca* ATCC 8724	6.5	9	6.5	6.5	6	6.5	9	9
*Shigella boydii* ATCC 9204	nt	11	nt	nt	nt	nt	nt	12
*Pseudomonas aeruginosa* ATCC 9027	nt	6	nt	nt	nt	nt	nt	6
*Escherichia coli*	6.5	6	6.5	6	6	6	6	6
*Salmonella enteridis*	6	6	6	6	7	6	6	7
Gram-positive bacteria								
*Bacillus cereus* ATCC 13061	7.5	8	10	9	8.5	9	9	11
*Staphylococcus aureus* ATCC 11632	12	11	13	12.5	13	12	12	13.5
*Streptococcus pneumoniae* ATCC 6301	13	13.5	15	13	16	14	13	15
Yeasts								
*Candida albicans*	6	6	6	6	6.5	6	6	6.5
*Candida membranaefaciens* ATCC 201377	nt	13	nt	nt	nt	nt	13	9
*Cryptococcus neoformans* ATCC 76484	nt	9	nt	nt	nt	nt	9	8
*Trichosporon mucoides* ATCC 204094	nt	10	nt	nt	nt	nt	10	9

^a^Each test was run in triplicate and the mean values are given; the solvent (methanol) was used for negative control

^b^Concentration of crude methanol extract: 1 mg/mL, 10 µL solution/6 mm disc; Ø < 7 mm: inactive, 7 mm ≤ Ø < 8 mm: slightly active, 8 mm ≤ Ø < 9 mm: significantly active, Ø ≥ 9 mm: very active; *nt* not tested

### *Sargassum incisifolium*

**Fig. 2:**
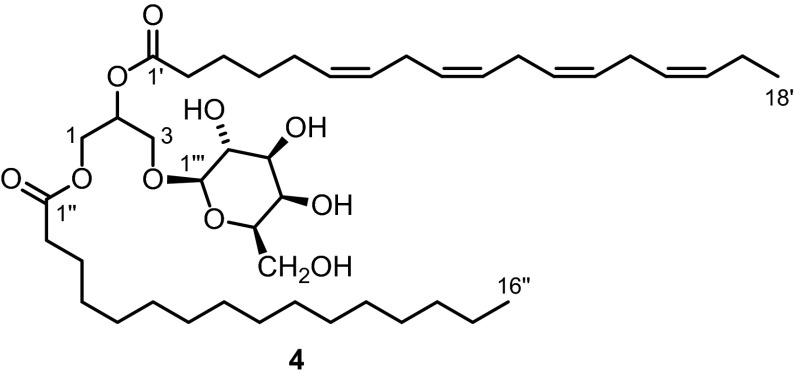
Structure of 1-*O*-palmitoyl-2-*O*-stearidonoyl-3-*O*-β-D-galactopyranosylglycerol (**4**)

The brown alga *Sargassum incisifolium* was extracted with ethyl acetate. The ethyl acetate extract was adsorbed on silica gel and subjected to flash chromatography with diethyl ether and then methanol. Purification of the diethyl ether fraction by column chromatography afforded fucosterol (**3**). The methanol fraction was subjected to normal-phase column chromatography to give two fractions. Fraction 2 afforded a mixture of inseparable monogalactosyldiacylglycerols bearing fatty acid side chains with only few olefinic groups according to the intensity of vinylic signals in the ^1^H NMR spectrum. Fraction 1 was further purified by reversed-phase HPLC using THF–H_2_O (50:50–80:20) as mobile phase to afford 1-*O*-palmitoyl-2-*O*-stearidonoyl-3-*O*-β-D-galactopyranosylglycerol (**4**) (Fig. 2) as an amorphous gum. The [M + NH_4_]^+^ peak at *m/z* = 768.7 and the [M + NH_4_ + Na – H]^+^ peak at *m/z* = 790.6 in the ESI-mass spectrum of **4** in combination with the number and intensity of ^1^H and ^13^C NMR signals led to the molecular formula C_43_H_74_O_10_. According to ESI mass spectrometry minor amounts of several other monogalactosyldiacylglycerols bearing fatty acids with a lower degree of unsaturation are present in the mixture. This corresponds to findings of Marcolongo et al. who investigated the monogalactosyldiacylglycerol fraction isolated from the thermophilic blue-green alga *Phormidium* sp. ETS-05 and identified palmitic acid and stearidonic acid as main acyl components during their fatty acid analysis by GC [26].

The IR spectrum of **4** exhibited absorption bands corresponding to hydroxyl (3391 cm^−1^) and carboxylic ester (1738 cm^−1^) groups. The 1D and 2D NMR spectra of **4** displayed signals for a carbon chain bearing four methylene-interrupted double bonds, a saturated carbon chain, a sugar moiety, a glycerol unit, and two carbonyl groups. The sugar residue could be identified as β-D-galactose by comparison of the ^13^C NMR shifts with those of various sugars listed in Ref. [27] and the typical proton coupling pattern. Most characteristic is the small coupling constant (*J* = 3.0 Hz) observed for the equatorial proton H-4′′′ at *δ* = 4.03 ppm which indicates that both neighboring protons are in axial positions (*cis* relationship). The sugar moiety is attached to the glycerol core at the terminal position C-3 based on the HMBC correlations of the protons at C-3 at *δ* = 3.76 (dd, *J* = 11.1, 6.2, 1 H, H-3b) and 3.92 ppm (dd, *J* = 11.3, 5.6 Hz, 1 H, H-3a) with the carbon C-1′′′ resonating at *δ* = 103.98 ppm (see Fig. S2a, center). This is also confirmed by NOESY correlations of the protons H-3a and H-3b with H-1′′′ (see Fig. S4, S4b).

One of the fatty acid chains was identified as stearidonic acid (C18:4) containing four skipped double bonds. This was proven by the presence of ^1^H NMR signals for six methylene protons at *δ* = 2.77–2.84 ppm (m, 6 H, H_2_-8′, H_2_-11′ and H_2_-14′) located between the double bonds. Correlations between the methylene and olefinic protons in the C18:4 acyl chain were observed in the COSY and NOESY spectra. The methylene protons at *δ* = 2.34 ppm (t, *J* = 7.7 Hz, 2 H, H_2_-2′) in the C18:4 chain display a cross-peak with the carbonyl group at *δ* = 173.50 ppm (C-1′) in the HMBC spectrum (Fig. S2a, bottom). The attachment of the stearidonoyl group at the central C-2 atom of the glycerol core was established by HMBC correlations of the protons at *δ* = 5.29–5.44 ppm (H-2 and others) with the signal of the carbonyl group at *δ* = 173.50 ppm (C-1′). In total, all proton and carbon signals of the C18:4 acyl chain could be unambiguously assigned by their correlations in 2D NMR experiments (COSY, HMBC, HSQC, and NOESY; see Supplementary Material).

**Fig. 3:**
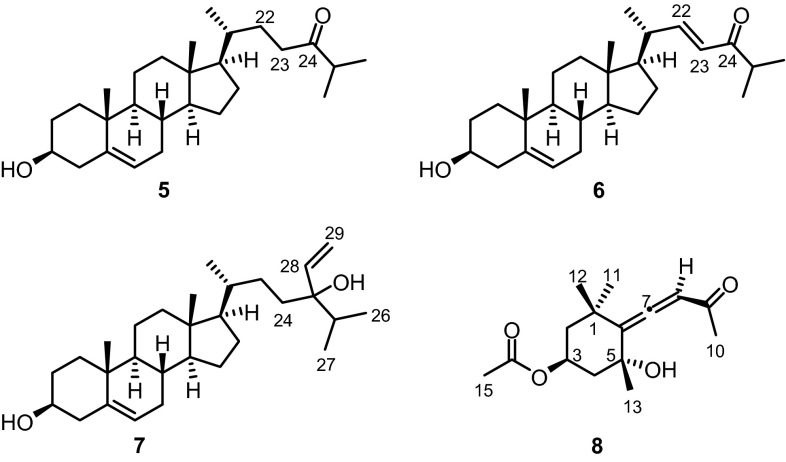
Structures of 24-ketocholesterol (**5**), (22*E*)-3β-hydroxycholesta-5,22-dien-24-one (**6**), saringosterol (**7**), apo-9′-fucoxanthinone (**8**)

The second acyl chain proved to be fully saturated as all olefinic proton and carbon signals could be assigned to the stearidonoyl part. The chain length of 16 carbon atoms was deduced from the intensities of the methylene signals in the ^1^H NMR spectrum and the molecular mass of 750 derived from the ESI mass spectrum. The proton signals of the first methylene unit at *δ* = 2.32 (t, *J* = 7.7 Hz, 2 H, H_2_-2′′) in this chain (C16:0) exhibited an HMBC correlation with the carbonyl carbon at *δ* = 173.78 ppm (C-1′′) which showed further HMBC cross-peaks with the methylene protons at *δ* = 4.22 (dd, *J* = 12.0, 6.4 Hz, 1 H, H-1a) and 4.40 ppm (dd, *J* = 12.0, 3.4 Hz, 1 H, H-1b) of the glycerol moiety (Fig. S2a). This result proved that the C16:0 acyl chain is attached at C-1 of the glycerol moiety.

Monogalactosyldiacylglycerols (MGDG) and digalactosyldiacylglycerols are widely spread in nature particularly in chloroplast membranes [28–31]. Accordingly, they have also been found in brown algae. For example, Kim et al. isolated four monogalactosyldiacylglycerols from the brown alga *S. thunbergii* collected at the coastal areas of Korea [32]. A series of monogalactosyldiacylglycerols has been identified by Liu and coworkers in *Sargassumhorneri* [33]. 1-*O*-Palmitoyl-2-*O*-stearidonoyl-3-*O*-β-D-galactopyranosylglycerol (**4**) has been mentioned as a trace component in a mixture of monogalactolipids obtained from *Scytonema* sp. as derived from enzymatic hydrolysis, HRMS, and EI-MS fragmentation studies [34]. A monogalactosyldiacylglycerol (MGDG) fraction that should contain considerable amounts of glycolipid **4** according to their fatty acid analysis by GC has been isolated by the group of Marcolongo from the thermophile blue-green alga *Phormidium* sp. ETS-05 [26]. The presence of the stearidonoyl side chain, however, was not supported by their ^1^H and ^13^C NMR data. The MGDG fraction displayed anti-inflammatory activity in croton-oil-induced ear edema and carrageenan-induced paw edema in mice [35], and on human articular cartilage [36, 37]. Herein, we describe the first isolation of 1-*O*-palmitoyl-2-*O*-stearidonoyl-3-*O*-β-D-galactopyranosylglycerol (**4**) from a brown alga (*Sargassumincisifolium*) with full assignment of the ^1^H and ^13^C NMR signals.

In the agar diffusion test, the crude extract of *Sargassum incisifolium* exhibited very strong antimicrobial activity against the Gram-positive bacteria *S. pneumoniae*, *S. aureus*, and *B. cereus* (Table 3). No activity was found against the Gram-negative bacteria, *Enterobacter cloacae*, *Klebsiella oxytoca*, *E. coli*, *S. enteridis*, and the yeast *C. albicans*. Moreover, the crude extract of *Sargassum incisifolium* shows antimalarial activity with an IC_50_ value of 57.80 ± 1.91 µg/mL for inhibition of the FCM29 strain of *Plasmodium falciparum*.

### Turbinaria ornata

The methanol extract of the marine brown alga *T.ornata* was extracted with dichloromethane. After column chromatography on silica gel, the four known steroids fucosterol (**3**) [13, 14] (Fig. 1), 24-ketocholesterol (**5**) (first preparation: Ref. [38], first isolation from an alga: Ref. [39]) (Fig. 3), (22*E*)-3β-hydroxycholesta-5,22-dien-24-one (**6**) (first isolation: Ref. [40], preparation: Ref. [41]), and saringosterol (**7**) (first isolations: Ref. [42, 43], synthesis: e.g., Ref. [21, 44, 45]) were isolated together with apo-9′-fucoxanthinone (**8**) (first report: Ref. [45], first isolation: Ref. [47]).

The mixture of 24-ketocholesterol (**5**) and enone **6** was obtained as a colorless, amorphous powder. The two components could be detected by GC–MS (**5**/**6** = 3:1). The respective EI mass spectra showed molecular ion peaks at *m/z* = 400 and at *m/z* = 398 which could be assigned to the molecular formulae C_27_H_44_O_2_ and C_27_H_42_O_2_, respectively. In the ^1^H and ^13^C NMR spectra the signals for the steroidal backbone are identical. Only the signals for the side chain could be differentiated and assigned to **5** or **6**, respectively. The most significant differences are the signals for C-22/C-23 and H-22/H-23. Compound **6** displayed the typical chemical shifts for olefinic protons and carbon atoms at these positions [*δ*_H_ = 6.08 (d, *J* = 15.4 Hz, 1 H, H-23), 6.73 (dd, *J* = 15.4, 8.7 Hz, 1 H, H-22); *δ*_C_ = 126.2 (C-23), 152.5 (C-22)]. Comparison of the ^1^H and ^13^C NMR data with those reported in the literature (**5**: Ref. [48], **6**: Ref. [40, 49]) confirmed the identity of compounds **5** and **6**. While numerous papers are available on the isolation of **5** from various kinds of organisms, enone **6** has been mentioned less frequently and has been found only in marine organisms.

Saringosterol (**7**) was obtained as a colorless, amorphous solid. Beside the signals for the steroidal backbone including the methyl groups, the ^1^H NMR spectrum revealed the presence of an additional double bond by three additional signals for olefinic protons. Signal doubling of the olefinic protons H-29 [*δ*_H_ = 5.12 (dd, *J* = 10.9, 1.5 Hz, 1 H, H-29a), 5.13 (dd, *J* = 10.9, 1.5 Hz, H-29a), 5.17 (dd, *J* = 17.3, 1.5 Hz, H-29b), 5.18 (dd, *J* = 17.3, 1.5 Hz, H-29b)] and the carbon atoms C-28 (*δ*_C_ = 142.55, 142.46) and C-29 (*δ*_C_ = 112.93, 112.84) indicated the presence of a 1:1 mixture of C-24 epimers. The ^1^H NMR data of the side chain are in agreement with those reported by Djerassi et al. [21]. The respective ^13^C NMR shifts have been compared with those published by the groups of Ayyad [45] and Wang [50]. In general a good agreement has been observed. The signals for C-28 and C-29 reported by the group of Ayyad are about 4–5 ppm upfield shifted compared to the values of Wang and us.

Saringosterol (**7**) has been isolated from diverse organisms, in particular brown algae (for examples see Ref. [42, 43, 50–54]). A variety of bioactivities has been disclosed for this compound including antitrypanosomal activity [54], selective LXRβ agonist activity (potential cholesterol reducing agent) [55], and inhibition of *Mycobacteriumtuberculosis* growth [56].

Compound **8** was isolated as an amorphous solid with a melting point of 84–85 °C and a specific optical rotation of [α]_D_^20^ = –18.0 (c = 0.05, MeOH). The molecular formula was determined as C_15_H_22_O_4_ from the [M + NH_4_]^+^ ion at *m/z* = 284 in the ESI–MS in combination with the number and intensities of the ^1^H and ^13^C NMR signals. Compound **8** shows a characteristic IR absorption band at ν = 1937 cm^−1^ indicating the presence of an allene group. The ^1^H NMR spectrum displayed signals for 21 protons which could be assigned to five methyl, two methylene and two methine groups according to the DEPT data. The ^13^C NMR spectrum displayed resonances for 15 carbon atoms including two carbonyl groups at *δ* = 196.63 and 170.34 ppm in CDCl_3_ and at *δ* = 200.86 and 172.41 ppm in CD_3_OD and two oxygenated carbon atoms at *δ* = 72.05 and 67.37 ppm in CDCl_3_ and 72.38 and 69.13 ppm in CD_3_OD (Table 4). The ^13^C NMR (CDCl_3_) signals for the carbon atoms of the allene moiety appear at *δ* = 118.45 (C-6), 209.47 (C-7), and 100.91 ppm (C-8). Comparison of the NMR data with those reported for apo-9′-fucoxanthinone (**8**) by Kobayashi et al. confirmed the identity of the compound (Table 4) [47]. Apo-9′-fucoxanthinone (**8**) was first reported as an oxidative degradation product of fucoxanthin [46, 57]. The structure and the absolute configuration of **8** were established by synthesis and X-ray crystallographic analysis [58]. Apo-9′-fucoxanthinone (**8**) has been isolated, for example, from the cultured marine dinoflagellate *Amphidinium* sp. [46] and from the brown algae *Scytosiphon lomentaria* [59], *S.thunbergii* [60], and *Cladostephusspongiosus* f. *verticillatus* [61]. This is the first report on the isolation of **8** from *T. ornata.* It has been disclosed previously that apo-9′-fucoxanthinone (**8**) exhibits cytotoxicity against murine lymphoma L-1210 and human epidermoid carcinoma KB cells in vitro with IC_50_ values of 0.29 and 0.24 µg/mL, respectively [47].

**Table 4:** ^1^H and ^13^C NMR spectroscopic data of apo-9′-fucoxanthinone (**8**) in CD_3_OD

Position	*Turbinaria ornata*		*Amphidinium* sp. [47]		*Turbinaria ornata*	*Amphidinium* sp. [47]
	*δ*_H_ (600 MHz)	*J* (Hz)	*δ*_H_	*J* (Hz)	*δ*_C_ (150 MHz)	*δ*_C_
1					37.11	37.2
2 ax eq	1.56 dd 2.00 ddd	12.8, 11.6 12.8, 4.1, 2.2	1.60 dd 2.04 ddd	12.7, 11.5 12.7, 4.1, 2.2	46.28	46.4
3	5.38 tt	11.6, 4.1	5.42 tt	11.5, 4.1	69.13	69.2
4 ax eq	1.46 br t 2.23 ddd	12.1 12.8, 4.1, 2.2	1.49 dd 2.27 ddd	12.7, 11.5 12.7, 4.1, 2.2	46.19	46.3
5					72.38	72.4
6					119.81	119.9
7					211.56	211.6
8	5.86 s		5.90 s		101.41	101.5
9					200.86	200.9
10	2.20 s		2.24 s		26.81	26.9
11	1.43 s		1.46 s		29.40	29.5
12	1.17 s		1.20 s		32.22	32.3
13	1.39 s		1.43 s		30.74	30.8
14					172.41	172.5
15	2.03 s		2.07 s		21.34	21.4

In the agar diffusion test the crude extract of *T.ornata* showed very strong antimicrobial activity against the Gram-negative bacteria *Shigella boydii*, *Enterobacter cloacae*, and *Klebsiella oxytoca* and the Gram-positive bacteria *S. pneumoniae*, *S. aureus*, and *B. cereus* (Table 3). An inhibiting activity was also detected against the yeasts *Candida membranaefaciens*, *Trichosporon mucoides*, and *Cryptococcus neoformans*.

### *Sargassum polycystum, Sargassum* sp. (*S. sect. Binderiana*), *Turbinaria decurrens, Turbinaria conoides,* and *Hormophysa cuneiformis*

Bioassay-guided fractionation of the methanol extracts of *Sargassum polycystum*, *Turbinaria decurrens*, *Turbinaria conoides*, and *Hormophysa cuneiformis* led in all cases to the isolation of fucosterol (**3**). Also the crude extract of *Sargassum* sp. (S. sect. Binderiana) contained fucosterol (**3**) as major component, but in this case it was obtained as an inseparable mixture with β-sitosterol.

The crude methanol extracts of *S. polycystum* and *Sargassum* sp. (S. sect. Binderiana) exhibited very strong antimicrobial activities against Gram-positive bacteria, in particular against *S. pneumoniae* and *S. aureus*, and less pronounced against *B. cereus* (Table 3). The crude methanol extract of *T. decurrens* was very active against the Gram-negative bacterium *Klebsiellaoxytoca*, the Gram-positive bacteria *S. pneumoniae*, *S. aureus*, and *B.cereus*, and against the yeasts *C.membranaefaciens*, *Trichosporonmucoides*, and *Cryptococcusneoformans*. Moreover, significant activity was observed against *Enterobacter cloacae*. Kumar et al. reported various antimicrobial and cytotoxic activities of different extracts of *T. conoides* (J. Agardh) Kutzing [62]. In our study, the crude methanol extract of *T. conoides* exhibited very strong activity against *S. pneumoniae*, *S. aureus*, and *B. cereus*. The crude methanol extract of *H. cuneiformis*, was very active against the Gram-positive bacteria *S. pneumoniae* and *S. aureus* and only slightly active against *B. cereus*.

Obviously, the crude methanol extracts of all species of brown algae investigated in the present study show a similar activity against various pathogenic microbes. In particular, in all cases a very strong activity against the Gram-positive bacteria *S. pneumoniae* and *S. aureus* was observed. This may be ascribed partly to the content of phytosterols, which have been isolated from all species of brown algae. The antibacterial activity of β-sitosterol, stigmasterol, and their acetates has been described earlier by Sharma [63]. Fucosterol (**3**) has been reported to show anti-oxidant and hepatoprotective activities in rats [64]. In addition, antihistaminic, anticholinergic, and antiviral activities have been described for fucosterol isolated from *T. conoides* (J. Agardh) Kutzing [65]. Saringosterol (**7**) has been identified as active principle in the extract of *Lessonia nigrescens* for the inhibition of *Mycobacteriumtuberculosis* [56].

## Experimental Section

### Plant Material

Samples of the brown algae *H. cuneiformis*, *T.ornata*, and *T. conoides*, were collected in October in Tamatave on the east coast of Madagascar. The marine algae of the species *S. polycystum* (BOL 169761), *S. ilicifolium*, *Sargassum* sp. (S. sect. Binderiana) (BOL 169760), and *T.decurrens* were collected in October in Beravy, located in Tuléar on the south-west coast of Madagascar. The brown alga *Sargassum incisifolium* (BOL 169752) was collected in May in Evatraha, a small village north of Fort-Dauphin located in the south-east of Madagascar.

The brown algae have been identified by Dr. Lydiane Mattio and Professor Robert J. Anderson, Biological Sciences Department and Marine Research Institute, University of Cape Town, South Africa. Voucher specimens of three species of brown algae investigated in this study have been deposited at the Bolus herbarium (BOL) of the University of Cape Town, South Africa. The corresponding BOL accession numbers are given in brackets after the name of the algae.

### Extraction and Isolation

General: The fresh seaweed was washed under tap water, rinsed with distilled water, subsequently dried at 48–50 °C using a universal drying oven (Binder FD 53, Germany), and then finely powdered in an Ultra Turrax Janke–Kunkel T25 S1 homogenizer (IKA, Germany) with a stitch of 1 mm. In all cases, except *Sargassumincisifolium* (extraction with ethyl acetate), we extracted the dried and crushed samples of the algae with methanol. When checking the methanol extract by TLC, we obtained different results concerning the polarity of the compounds. Depending on whether the compounds were less polar (best eluted with diethyl ether) or more polar (best eluted with dichloromethane), we performed a second extraction with either diethyl ether or dichloromethane, respectively. Following this procedure, we obtained the non-polar compounds of the methanol extract that were purified by column chromatography.

The crushed and dried material of *S. ilicifolium* (500 g) was extracted with methanol. After removal of the solvent in vacuo, the methanol extract (11 g) was further extracted with different solvents of increasing polarity, namely diethyl ether, dichloromethane, and ethyl acetate. The successive extractions were carried out under magnetic stirring at room temperature. After removal of the solvent in vacuo, the diethyl ether extract (1.6 g) was subjected to flash chromatography on silica gel using pentane–diethyl ether (3:2) as eluent. A fraction of 130 mg was obtained, which was further purified by column chromatography (eluent: pentane–diethyl ether, 10:1) followed by preparative TLC (pentane–diethyl ether 10:1) to afford 2.0 mg of fucosterol (**3**). The dichloromethane extract (3.0 g) was separated into two fractions by column chromatography on silica gel using pentane–diethyl ether (3:2) as eluent. Fraction 1 (118 mg) was subjected to another column chromatographic separation on silica gel using pentane–diethyl ether (3:2) as eluent to afford two subfractions (A and B). Subfraction A (76 mg) was further purified by column chromatography on silica gel using pentane–dichloromethane (1:1) as mobile phase to afford 10 mg of 1,1′,2-trinorsqualenol (**1**). Subfraction B (15 mg) was further purified by column chromatography eluting with pentane–dichloromethane (9:1) to obtain 8.0 mg of stigmasta-5,28-dien-3β-ol (**2**). Fraction 2 (300 mg) was subjected to column chromatography on silica gel with pentane–diethyl ether (3:2) and further purified by a second column chromatographic separation with pentane–ethyl acetate (7:3) to give 3 mg of stigmasta-5,28-dien-3β-ol (**2**). The ethyl acetate extract (563 mg) was subjected to flash chromatography on silica gel to afford one main fraction (70 mg). Purification of this fraction by column chromatography on silica gel followed by preparative TLC afforded 3 mg of fucosterol (**3**).

The crushed material of *Sargassum incisifolium* (80 g) was exhaustively extracted with ethyl acetate to afford 2.0 g of a crude product. The ethyl acetate extract (2.0 g) was subjected to flash chromatography on silica gel using diethyl ether and subsequently methanol as eluents. The fraction eluting with diethyl ether (1.0 g) was separated by column chromatography on silica gel using pentane–diethyl ether (3:1) to afford a main fraction of 310 mg which was further purified by column chromatography using pentane–diethyl ether (7:3) as eluent to afford 180 mg of fucosterol (**3**). The fraction eluting with methanol (1.0 g) was subjected to flash chromatography on silica gel with ethyl acetate–methanol (10:1) to obtain 500 mg of a product mixture. Subsequent column chromatography on silica gel with ethyl acetate as eluent afforded two fractions of 40 mg (first fraction) and 70 mg (second fraction). Both fractions were subjected to column chromatography on silica gel using ethyl acetate–methanol (20:1) as mobile phase. The first fraction (20 mg) was further purified by preparative HPLC (column: Vydac 208TP1030, reversed-phase C8, 30 × 250 mm; flow rate: 20 mL min^−1^; eluent A: H_2_O + 0.1 % TFA, eluent B: THF + 0.1 % TFA; gradient from 50 to 80 % B in 20 min) to yield 6 mg of the monogalactosyldiacylglycerol **4**. The second fraction afforded 5 mg of a mixture of monogalactosyldiacylglycerols.

The crushed material of *T. ornata* (680 g) was extracted with methanol. The methanol extract was concentrated under reduced pressure and the residue (17 g) was further extracted with dichloromethane. After removal of the solvent in vacuo, the residue (9.0 g) was subjected to flash chromatography on silica gel using dichloromethane as eluent. The resulting mixture (260 mg) was further purified by column chromatography on silica gel eluting with pentane–diethyl ether (3:2) to obtain four fractions. Fraction 1 (19 mg) was identified as fucosterol (**3**). Fraction 2 (5.0 mg) showed one spot on the TLC but was identified as a mixture of the two steroids **5** and **6** in a ratio of about 3:1 according to GC–MS and ^1^H NMR spectroscopy. Fraction 3 (8.0 mg) was identified as saringosterol (**7**) and fraction 4 (19 mg) as apo-9′-fucoxanthinone (**8**).

The crushed and dried *S. polycystum* (3.4 g) was extracted with methanol. The organic extract was evaporated to dryness and a dark oily residue (85 mg) was obtained. The crude extract (85 mg) was then further extracted with dichloromethane and purified by column chromatography on silica gel using pentane–ethyl acetate (10:1) as mobile phase to afford 12 mg of fucosterol (**3**).

The crushed and dried plant material of *Sargassum* sp. (S. sect. Binderiana) (16 g) was minced and extracted exhaustively with methanol. After filtration, the organic extract was evaporated to dryness, and a dark oily residue was obtained. The crude extract was further extracted with diethyl ether. The diethyl ether extract (900 mg) was subjected to flash chromatography on silica gel using pentane–diethyl ether (3:2) to give two fractions. Fraction 1 contained 2-(*tert*-butyl)-4-chloro-5-methylphenol as an artifact and was disposed. Fraction 2 (78 mg), was separated by column chromatography on silica gel using pentane–ethyl acetate (9:1) to give 6.0 mg of a mixture of fucosterol (**3**) and β-sitosterol in a ratio of 1:1.3.

The crushed material of *T. decurrens* (12 g) was repeatedly extracted with methanol and the combined extracts were concentrated under reduced pressure. The crude extract (300 mg) was subjected to column chromatography on silica gel with a mixture of pentane–diethyl ether (4:1) to give 19 mg of a product mixture which was further purified by a second column chromatography using the same conditions to afford 13 mg of fucosterol (**3**).

The crude extract of *T. conoides* was obtained after extraction of the crushed material (40 g) with methanol. After removal of the solvent, the residue (960 mg) was extracted with dichloromethane at room temperature. The dichloromethane extract (890 mg) was subjected to flash chromatography on silica gel with diethyl ether. The diethyl ether fraction (190 mg) was separated by column chromatography on silica gel using pentane–diethyl ether (4:1) as eluent to provide 47 mg of a product mixture. Another purification step by column chromatography on silica gel with pentane–diethyl ether (4:1) as mobile phase afforded 21 mg of fucosterol (**3**).

The dried plant material (9.4 g) of *H. cuneiformis* was minced and extracted exhaustively with methanol. After evaporation of the solvent, a dark oily residue (84 mg) was obtained. The crude extract (84 mg) was then repeatedly extracted with diethyl ether, and the combined diethyl ether extracts were concentrated under reduced pressure to give a residue of 65 mg. The diethyl ether extract (65 mg) was separated by column chromatography on silica gel using a mixture of pentane–ethyl acetate (4:1) as eluent to afford 1 mg of fucosterol (**3**).

### Spectroscopic Characterization

General: Optical rotations were determined on a Perkin Elmer 341 polarimeter at a wavelength of 589 nm (sodium D line) using a 1.0-decimeter cell with a total volume of 1.0 mL. UV spectra were measured on a Perkin Elmer Lambda 25 UV–Vis spectrometer. Fluorescence spectra were measured on a Varian Cary Eclipse. IR spectra were recorded on a Thermo Nicolet Avatar 360 E. S. P. FT-IR spectrometer using the ATR technique (attenuated total reflectance). NMR spectra were recorded on a Bruker AVANCE III 600 spectrometer. The chemical shifts *δ* are reported in ppm using the solvent signal as internal standard. Assignment of the ^1^H NMR and ^13^C NMR signals was achieved using the following 2D NMR experiments: COSY, HSQC, HMBC, NOESY, and HSQC-TOCSY. The mass spectra were measured by GC–MS coupling with an Agilent Technologies 6890 N GC system equipped with a 5973 N Mass Selective Detector (electron impact, 70 eV). ESI–MS were recorded on a Bruker–Esquire mass spectrometer with an ion trap detector; positive and negative ions were detected. Thin layer chromatography was performed on aluminum plates coated with silica gel 60-F_254_ (Merck). For visualization, the plates were analyzed under UV light or treated with a solution of 0.5 g vanillin dissolved in 100 mL of 80/20 (v/v) sulfuric acid/ethanol and subsequently heated. Analytical HPLC was carried out on an Agilent 1100 device equipped with a G1315B UV-DAD (detection at 215, 260 and 560 nm), G1321A fluorescence and an evaporative light scattering detector (ELS 1000, Polymer Laboratories) using a Vydac 208TP104 column (reversed-phase C8, 4.6 × 250 mm) under the following conditions: flow rate: 1.0 mL min^−1^; eluent A: H_2_O + 0.1 % TFA; eluent B: MeCN + 0.1 % TFA; gradient from 20 to 90 % B in 35 min. Preparative HPLC was carried out using a Varian PrepStar system with a Varian ProStar Model 320 UV and an evaporative light scattering detector (ELS 1000, Polymer Laboratories) connected via a Sunchrom Quick-Split splitter.

(4*E*,8*E*,12*E*,16*E*)-4,8,13,17,21-Pentamethyldocosa-4,8,12,16,20-pentaen-1-ol (1,1′,2-trinorsqualenol) (**1**): Colorless oil; IR (ATR): ν (cm^−1^) = 3345 (br), 2922, 2854, 1667, 1440, 1378, 1153, 1056, 972, 900, 838, 740; ^1^H NMR (600 MHz, CDCl_3_): see Table 1; ^13^C NMR and DEPT (150 MHz, CDCl_3_): see Table 2; GC–MS (EI, 70 eV): *m/z* (%) = 386 (3), 343 (2), 317 (4), 163 (5), 149 (12), 136 (14), 121 (18), 95 (98), 81 (63), 69 (100), 55 (20), 41 (29); ESI–MS (25 V): *m/z* = 404.4 [M + NH_4_]^+^.

Stigmasta-5,28-dien-3β-ol (**2**) (1:1 mixture of C-24 epimers): Amorphous solid; mp **=** 119–120 °C (lit., 1:1 mixture of C-24-epimers: mp = 123–127 °C [23], (24*R*)-epimer: mp = 132–133 °C [21], (24*S*)-epimer: mp = 141–142 °C [21]); ^1^H NMR (600 MHz, CDCl_3_, selected signals): *δ* (ppm) = 0.69 (s, 3 H, H-18), 0.85–0.91 (m, 6 H, H_3_-26, H_3_-27, two epimers), 1.02 (s, 3 H, H_3_-19), 3.50–3.57 (m, 1 H, H-3), 5.17 (dd, *J* = 17.9, 1.4 Hz, 1 H, H-29a), 5.29 (ddd, *J* = 11.3, 2.6, 1.4 Hz, 1 H, H-29b), 5.35–5.38 (m, 1 H, H-6), 5.72–5.79 (m, 1 H, H-28, two epimers); ^13^C NMR (150 MHz, CDCl_3_, selected signals): *δ* (ppm) = 16.39, 16.45, 17.48, 17.50 (C-26, C-27, two epimers), 71.59 (C-3), 116.19 (C-29), 121.50 (C-6), 136.86, 136.95 (C-28, two epimers), 140.90 (C-5, derived from HMBC spectrum).

1-*O*-Palmitoyl-2-*O*-stearidonoyl-3-*O*-β-D-galactopyranosylglycerol (**4**): Amorphous gum; mp = 98–99 °C; IR (ATR): ν (cm^−1^) = 3391 (br), 2923, 2853, 1738, 1163, 1071; ^1^H NMR (600 MHz, CDCl_3_): *δ* (ppm) = 0.89 (t, *J* = 7.2 Hz, 3 H, H_3_-16′′), 0.98 (t, *J* = 7.5 Hz, 3 H, H_3_-18′), 1.22–1.35 (m, 24 H, H_2_-4′′–H_2_-15′′), 1.37–1.43 (m, 2 H, H_2_-4′), 1.57–1.67 (m, 4 H, H_2_-3′, H_2_-3′′), 2.04–2.12 (m, 4 H, H_2_-5′, H_2_-17′), 2.32 (t, *J* = 7.7 Hz, 2 H, H_2_-2′′), 2.34 (t, *J* = 7.7 Hz, 2 H, H_2_-2′), 2.77–2.84 (m, 6 H, H_2_-8′, H_2_-11′, H_2_-14′), 2.93 (br s, 1 H, OH), 3.05 (br s, 1 H, OH), 3.56 (t, *J* = 4.9 Hz, 1 H, H-5′′), 3.61 (dd, *J* = 9.9, 3.4 Hz, 1 H, H-3′′′), 3.65–3.68 (m, 1 H, H-2′′′), 3.76 (dd, *J* = 11.1, 6.2 Hz, 1 H, H-3a), 3.86–3.90 (m, 1 H, H-6′′′a), 3.92 (dd, *J* = 11.3, 5.6 Hz, 1 H, H-3b), 4.00 (dd, *J* = 12.2, 6.2 Hz, 1 H, H-6′′′b), 4.03 (d, *J* = 3.0 Hz, 1 H, H-4′′′), 4.22 (dd, *J* = 12.0, 6.4 Hz, 1 H, H-1a), 4.29 (dd, *J* = 7.5, 1.1 Hz, 1 H, H-1′′′), 4.40 (dd, *J* = 12.0, 3.4 Hz, 1 H, H-1b), 5.29–5.44 (m, 9 H, H-2, H-6′, H-7′, H-9′, H-10′, H-12′, H-13′, H-15′, H-16′); ^13^C NMR and DEPT (150 MHz, CDCl_3_): *δ* (ppm) = 14.12 (C-16′′), 14.27 (C-18′), 20.55 (C-17′), 22.69 (C-15′′), 24.48 (C-3′), 24.87 (C-3′′), 25.52, 25.61 (2 C) (C-8′, C-11′, C-14′), 26.82 (C-5′), 28.99 (C-4′), 29.05–29.76 (C-4′′–C-13′′), 31.92 (C-14′′), 34.14 (C-2′′), 34.28 (C-2′), 62.68 (C-1), 63.01 (C-6′′′), 68.46 (C-3), 69.55 (C-4′′′), 70.18 (C-2), 71.74 (C-2′′′), 73.44 (C-3′′′), 74.51 (C-5′′′), 103.98 (C-1′′′), 127.10 (C-15′), 127.89, 128.08, 128.16, 128.23, 128.30 (C-7′, C-9′, C-10′, C-12′, C-13′), 129.52 (C-6′), 131.97 (C-16′), 173.50 (C-1′), 173.78 (C-1′′); ESI–MS (+25 V): *m/z* = 768.7 [M + NH_4_]^+^, 790.6 [M + NH_4_ + Na − H]^+^; ESI–MS (+75 V): *m/z* = 773.7 [M + Na]^+^, 795.7 [M + 2Na − H]^+^; ESI–MS (−10 V): *m/z* = 749.4 [M–H]^−^, 771.4 [M + Na − 2H]^−^. 2D-NMR spectra (COSY, HMBC, HSQC, NOESY, and DOSY) of compound **4**: see Supplementary Material.

24-Oxocholest-5-en-3β-ol (24-ketocholesterol) (**5**) (3:1 mixture with **6**): Colorless amorphous solid; ^1^H NMR (600 MHz, CDCl_3_, selected signals): *δ* (ppm) = 0.93 (d, *J* = 6.8 Hz, 3 H, H_3_-21), 1.10 (d, *J* = 6.8 Hz, 6 H, H_3_-26, H_3_-27), 1.24–1.28^*^ (m, 1 H, H-22a), 1.37–1.43^*^ (m, 1 H, H-20), 1.70–1.78^*^ (m, 1 H, H-22b), 2.36–2.40 (m, 1 H, H-23a), 2.44–2.50 (m, 1 H, H-23b), 2.64 (sept, *J* = 7.1 Hz, 1 H, H-25), ^*^signals determined from HSQC spectrum; ^13^C NMR (150 MHz, CDCl_3_, selected signals): *δ* (ppm) = 18.31, 18.38 (C-26, C-27), 18.51 (C-21), 29.84 (C-22), 35.39 (C-20), 37.24 (C-23), 40.83 (C-25), 215.48 (C-24); GC–MS (EI, 70 eV): *m/z* (%) = 400 (50), 382 (41), 314 (33), 271 (33), 255 (35), 213 (58), 145 (47), 105 (52), 43 (100).

(22*E*)-3β-Hydroxycholesta-5,22-dien-24-one (**6**) (1:3 mixture with **5**): ^1^H NMR (600 MHz, CDCl_3_, selected signals): *δ* (ppm) = 2.84 (sept, *J* = 6.8 Hz, 1 H, H-25), 6.08 (d, *J* = 15.4 Hz, 1 H, H-23), 6.73 (dd, *J* = 15.4, 8.7 Hz, 1 H, H-22); ^13^C NMR (150 MHz, CDCl_3_, selected signals): *δ* (ppm) = 126.2^*^ (C-23), 152.5^*^ (C-22), 204.5 (C-24), ^*^signals determined from HMBC spectrum; GC–MS (EI, 70 eV): *m/z* (%) = 398 (5), 380 (7), 309 (6), 271 (9), 255 (9), 187 (24), 159 (22), 126 (100).

Saringosterol (**7**) (1:1 mixture of C-24 epimers): Colorless amorphous solid; IR (ATR): ν (cm^−1^) = 3325 (br), 2959, 2931, 2865, 2850, 1667, 1459, 1431, 1375, 1055, 1021, 994, 957, 918, 798, 739; ^1^H NMR (600 MHz, CDCl_3_, selected signals): *δ* (ppm) = 5.12 (dd, *J* = 10.9, 1.5 Hz) and 5.13 (dd, *J* = 10.9, 1.5 Hz, H-29a, 1 H)^*^, 5.17 (dd, *J* = 17.3, 1.5 Hz) and 5.18 (dd, *J* = 17.3, 1.5 Hz, H-29b, 1 H)^*^, 5.33–5.35 (m, 1 H, H-6), 5.79 (dd, *J* = 17.7, 11.3 Hz) and 5.80 (dd, *J* = 17.3, 10.9 Hz, H-28, 1 H)^*^; ^13^C NMR and DEPT (150 MHz, CDCl_3_, selected signals): *δ* (ppm) = 71.80 (C-3), 112.84 and 112.93 (C-29)^*^, 121.70 (C-6), 140.73 (C-5), 142.46 and 142.55 (C-28)^*^. ^*^Two sets of signals due to the presence of two epimers.

Apo-9′-fucoxanthinone (**8**): Amorphous solid; mp = 84–85 °C (lit., mp = 76.0–78.5 °C) [66]; [α]_D_^20^ = −18.0 (c = 0.05, MeOH) [lit., [α]_D_^22^ = −23.0 (c = 0.04, MeOH) [67], [α]_D_^21^ = −29.7 (EtOH) [68], [α]_D_ = − 36.0 (c = 0.6) [66], [α]_D_^19^ = −284 (c = 0.1, MeOH) [47], [α]_D_^26^ = −25.8 (c = 0.1, CHCl_3_) [69]]; CD Δ*ε* MeOH (λ nm): −3.32 (206), +3.88 (229), −3.07 (257) [lit., CD Δ*ε* EtOH (λ nm): −3.27 (207), +3.31 (230), −3.13 (257)] [47]; UV (MeOH): λ (nm) = 228; fluorescence (MeOH): λ_max_ (228 nm) = 307 nm; IR (ATR): ν (cm^−1^) = 3433 (br), 2960, 2930, 2866, 2056, 1937, 1734, 1675, 1454, 1367, 1242, 1165, 1073, 1030, 958, 858, 818; ^1^H NMR (600 MHz, CDCl_3_): *δ* (ppm) = 1.15 (s, 3 H, H_3_-12), 1.40–1.46 (m, 1 H, H-2ax), 1.421 (s, 3 H), 1.424 (s, 3 H) (H_3_-11, H_3_-13), 1.52 (t, *J* = 12.8 Hz, 1 H, H-4ax), 2.02 (ddd, *J* = 12.8, 4.3, 2.1 Hz, 1 H, H-2 eq), 2.04 (s, 3 H, H_3_-15), 2.18 (s, 3 H, H_3_-10), 2.32 (ddd, *J* = 12.8, 4.3, 2.1 Hz, 1 H, H-4 eq), 5.35–5.41 (m, 1 H, H-3), 5.86 (s, 1 H, H-8); ^13^C NMR and DEPT (150 MHz, CDCl_3_): 21.33 (C-15), 26.41 (C-10), 28.93 (C-11), 30.74 (C-13), 31.61 (C-12), 36.02 (C-1), 44.98, 45.02 (C-2, C-4), 67.37 (C-3), 72.05 (C-5), 100.91 (C-8), 118.45 (C-6), 170.34 (C-14), 196.63 (C-9), 209.47 (C-7); ^1^H NMR (600 MHz, CD_3_OD) and ^13^C NMR (150 MHz, CD_3_OD): see Table 4; GC–MS (EI, 70 eV): *m/z* (%) = 266 (0.2) (M^+^), 251 (2), 206 (4), 191 (21), 163 (19), 149 (9), 131 (12), 123 (50), 107 (10), 91 (7), 43 (100). ESI–MS (10 V): *m/z* = 284 [M + NH_4_]^+^, 289 [M + Na]^+^, 555 [2 M + Na]^+^. Anal. calcd for C_15_H_22_O_4_: C 67.64, H 8.33; found: C 67.94, H 8.52.

### Biological Testing

Antimicrobial assay: The antimicrobial activities were determined using the agar diffusion technique in Petri dishes. The crude brown algal extracts were tested for their antimicrobial activity against three Gram-positive bacteria: *B. cereus* (ATCC 13061), *S. aureus* (ATCC 11632), and *S. pneumoniae* (ATCC 6301); six Gram-negative bacteria: *Enterobacter cloacae* (ATCC 700323), *Klebsiella oxytoca* (ATCC 8724), *Shigella boydii* (ATCC 9204)*, E. coli*, *S. enteridis, P. aeruginosa* (ATCC 9027); and against four yeast strains: *C. albicans, C. membranaefaciens* (ATCC 201377)*, Cryptococcus neoformans* (ATCC 76484), and *Trichosporon mucoides* (ATCC 204094). The pathogens were supplied by the Laboratoire de Microbiologie de l’Environnement (LME), Centre National de Recherche sur l’Environnement (CNRE), Antananarivo, Madagascar. The crude extracts were dissolved in methanol with a concentration of 1 mg/mL. A sample (10 μL) of each solution was added via a pipette onto a sterile antibiotic filter disc of 6 mm diameter and oven dried at 40–50 °C. The discs were placed on Müller–Hinton agar plates which had been inoculated with the microorganisms mentioned above. The plates were incubated for 24 h at 37 °C for the bacteria and for 48 h at 25 °C for the yeast. The diameters of the inhibition zones generated around the discs were measured (Ø in mm). The tests were performed in triplicate and the mean values were determined. Methanol, used to dissolve the extracts, was checked for the absence of antimicrobial activity. The diameters of the halos of inhibition can be rationalized on a qualitative basis as follows: Ø < 7 mm: inactive, 7 mm ≤ Ø < 8 mm: slightly active, 8 mm ≤ Ø < 9 mm: significantly active, Ø ≥ 9 mm: very active.

Antimalaria test: The antiplasmodial activity against the FCM29 strain of *Plasmodium falciparum* was determined by using the microfluorimetric assay previously reported [70]. The result is given as an IC_50_ value in µg/mL.

## Conclusions

We present one of the first extensive studies on the non-polar chemical constituents of brown algae from the coast of Madagascar. Our results confirm the early reports that fucosterol (**3**) is the major sterol of brown algae (Phaeophyceae) [14]. Moreover, the sterols stigmasta-5,28-dien-3β-ol (**2**), 24-ketocholesterol (**5**), (22*E*)-3β-hydroxycholesta-5,22-dien-24-one (**6**), and saringosterol (**7**) have been isolated from *S. ilicifolium* and *T.ornata*, respectively. The allenic norterpenoid apo-9′-fucoxanthinone (**8**) was obtained from *T.ornata*. For the first time, 1,1′,2-trinorsqualenol (**1**) has been obtained as a pure compound from natural sources and moreover, its isolation from a marine organism (*S. ilicifolium*) is unprecedented. The glycolipid 1-*O*-palmitoyl-2-*O*-stearidonoyl-3-*O*-β-D-galactopyranosylglycerol (**4**) was obtained from the brown alga *Sargassum incisifolium* and for the first time, we report the complete assignment of its ^1^H NMR and ^13^C NMR data. Antimicrobial tests of the crude extracts of the various brown algae revealed a strong activity against Gram-positive bacteria, in particular against *S. aureus* and *Streptococcus pneumonia*, which may ascribed to the content of phytosterols.

## Electronic supplementary material

Supplementary material 1 (PDF 1873 kb)
