# Supplementary material for: Brown Algae (Phaeophyceae) from the Coast of Madagascar: preliminary Bioactivity Studies and Isolation of Natural Products
Source: Nat Prod Bioprospect. 2015 Sep 10;5(5):223–35. doi: 10.1007/s13659-015-0068-0 (PMC4607676; doi:10.1007/s13659-015-0068-0)
Supplement: Supplementary file 1 — Supplementary material 1 (PDF 1873 kb) [file 13659_2015_68_MOESM1_ESM.pdf]

Supplementary Material

Brown Algae (Phaeophyceae) from the Coast of Madagascar: Preliminary Bioactivity Studies and Isolation of Natural Products

Marie Pascaline Rahelivao [1], Margit Gruner [1], Hanta Andriamanantoanina [2], Ingmar Bauer [1], Hans-Joachim Knölker [1,*]

[1] Department Chemie, Technische Universität Dresden, Bergstr. 66, 01069 Dresden, Germany

[2] Centre National de Recherche sur l'Environnement, MESupRes, BP 1739, Antananarivo 101, Madagascar

***** Author to whom correspondence should be addressed; E-Mail: hans-joachim.knoelker@tu-dresden.de; Tel.: +49-351-463-34659 ; Fax: +49-351-463-37030.

Table of Contents

**Figure S1**	COSY spectrum of compound **4**	2
**Figure S1a**	Details of the COSY spectrum of compound **4**	3
**Figure S2**	HMBC spectrum of compound **4**	4
**Figure S2a**	Characteristic correlations in the HMBC spectrum of compound **4**	5
**Figure S3**	HSQC spectrum of compound **4**	6
**Figure S4**	NOESY spectrum of compound **4**	7
**Figure S4a**	Details of the NOESY spectrum of compound **4**	7
**Figure S4b**	Details of the NOESY spectrum of compound **4**	8
**Figure S5**	DOSY spectrum of compound **4**	9

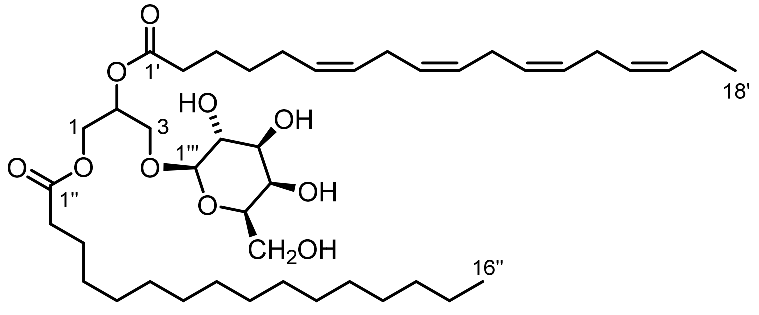

4

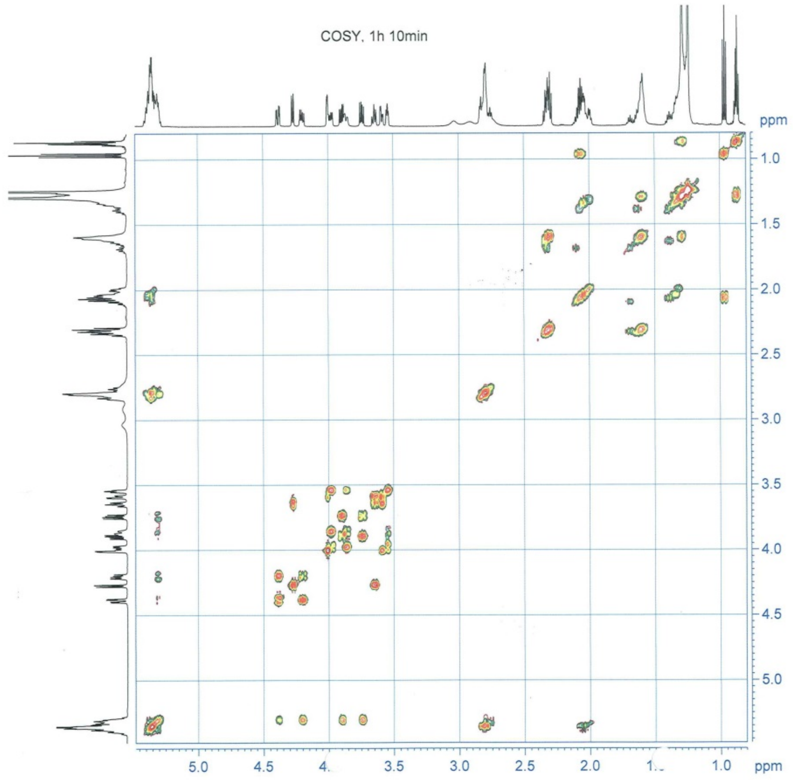

Fig. S1 COSY spectrum of compound **4**

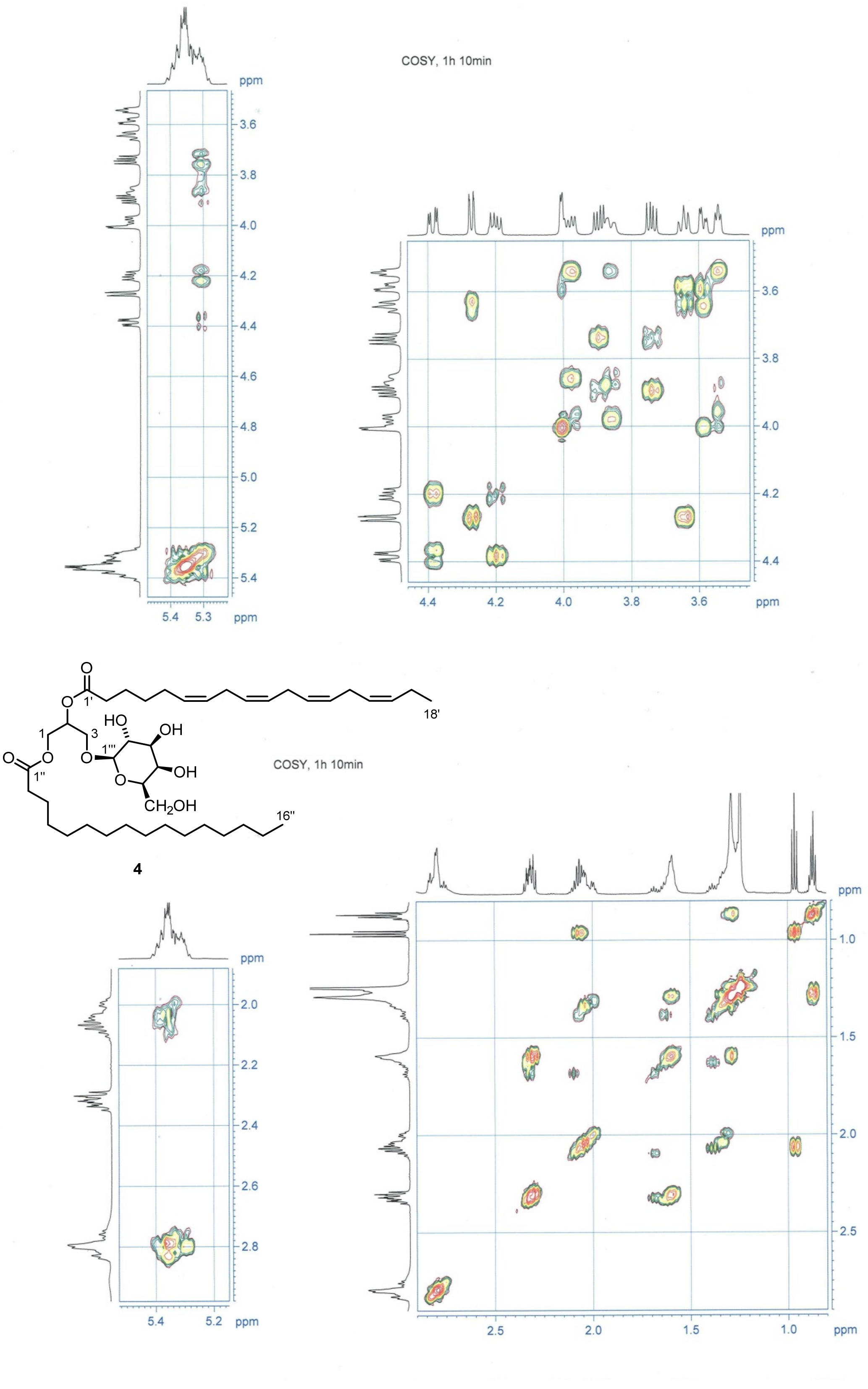

Fig. S1a Details of the COSY spectrum of compound **4**

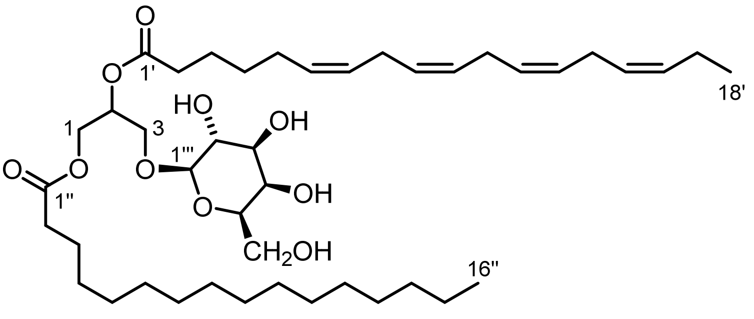

4

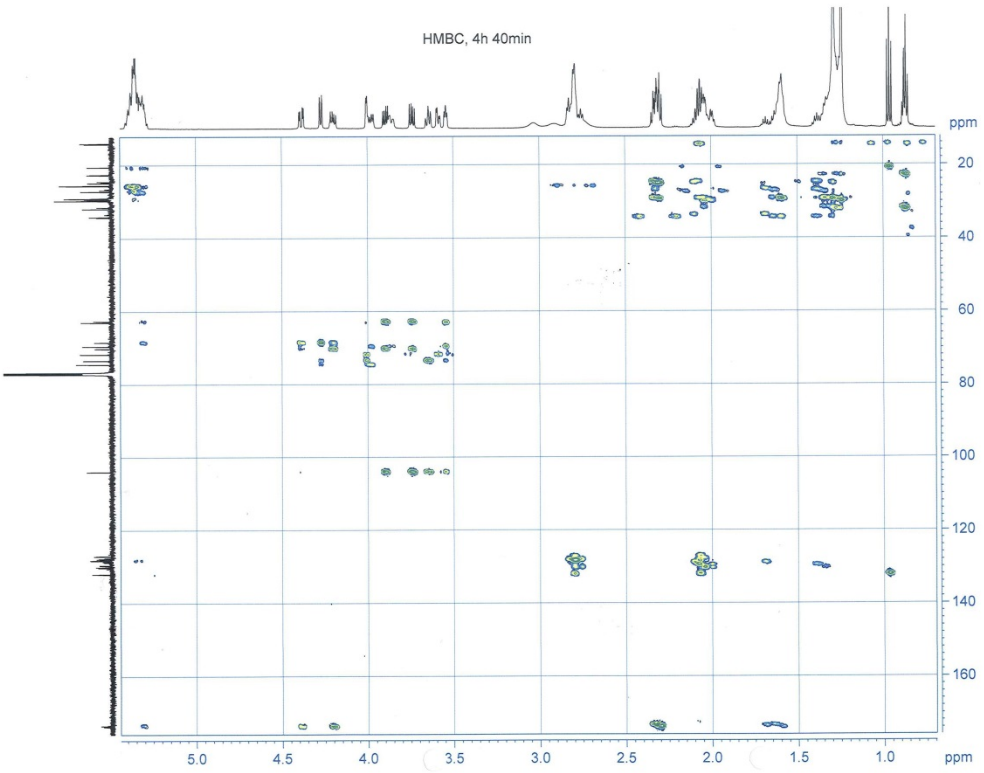

Fig. S2 HMBC spectrum of compound **4**

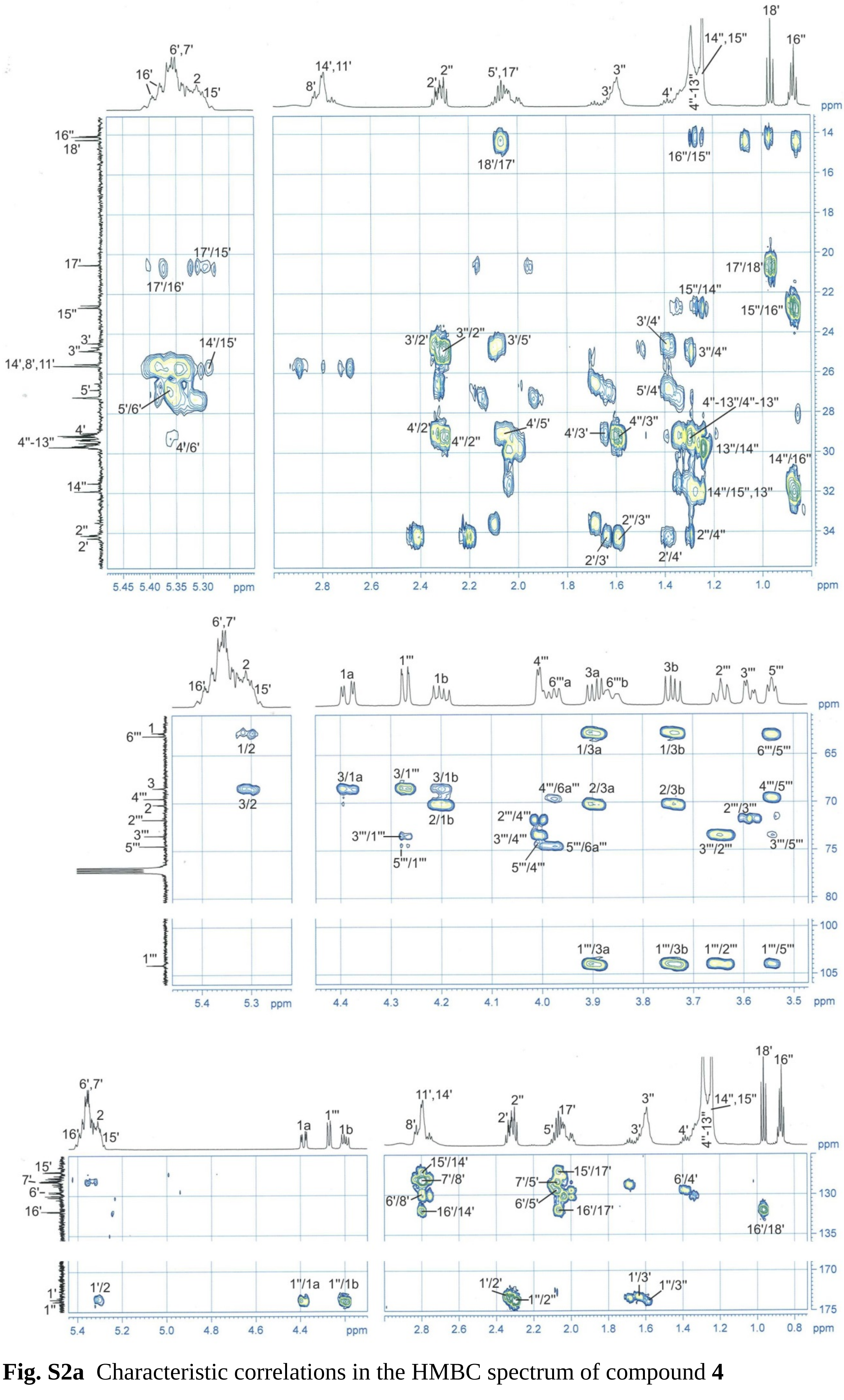

Fig. S2a Characteristic correlations in the HMBC spectrum of compound **4**

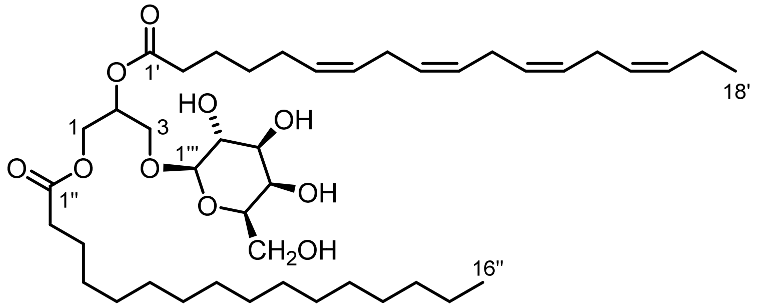

4

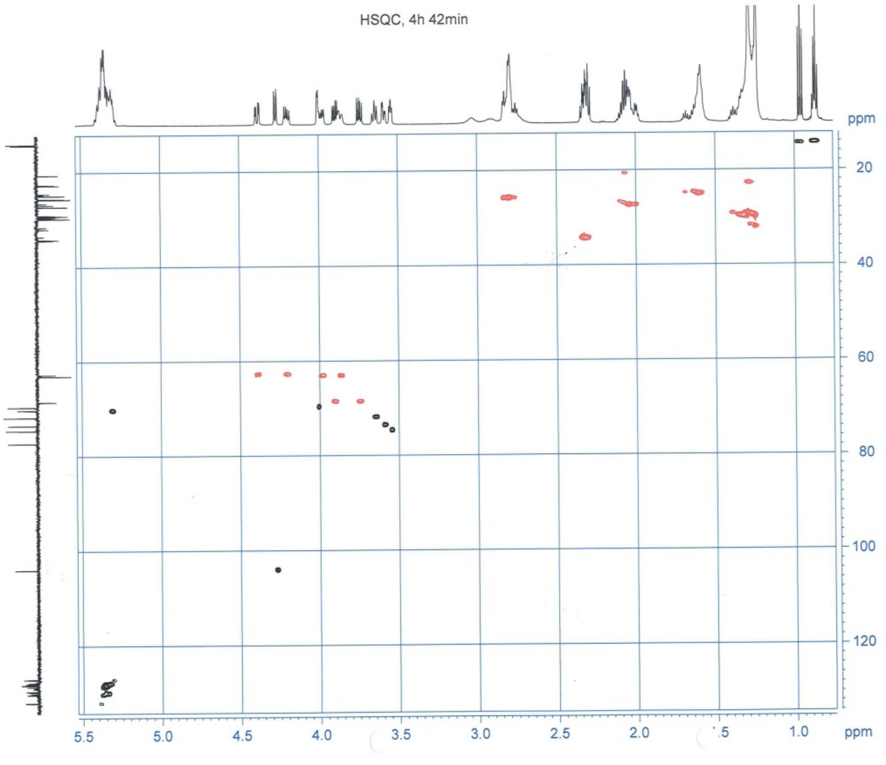

Fig. S3 HSQC spectrum of compound **4**

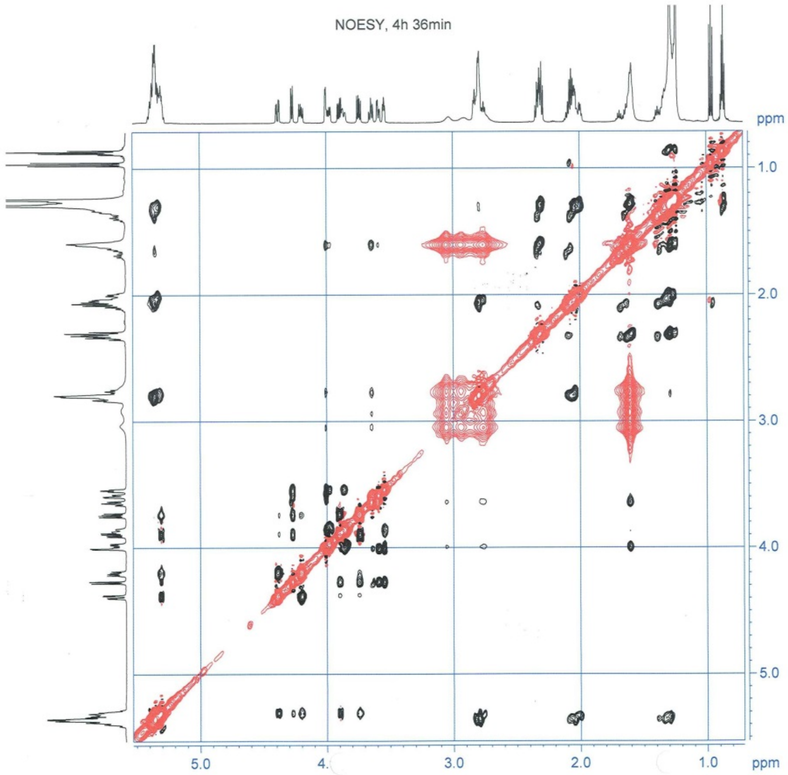

Fig. S4 NOESY spectrum of compound **4**

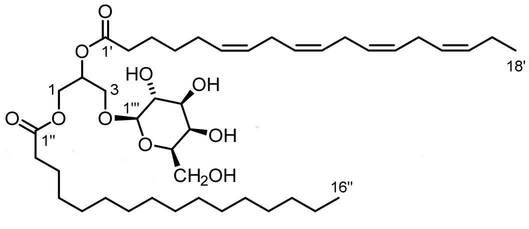

4

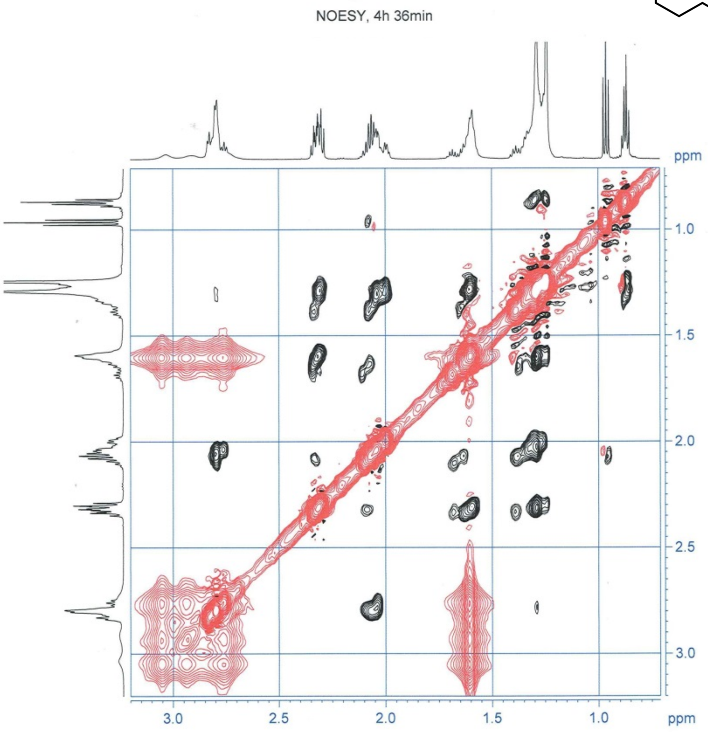

Fig. S4a Details of the NOESY spectrum of compound **4**

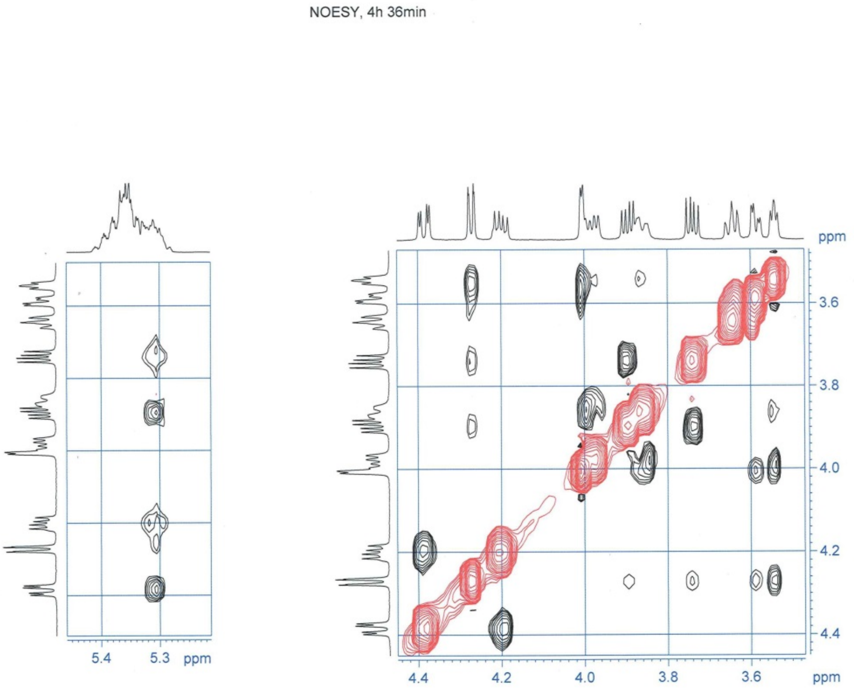

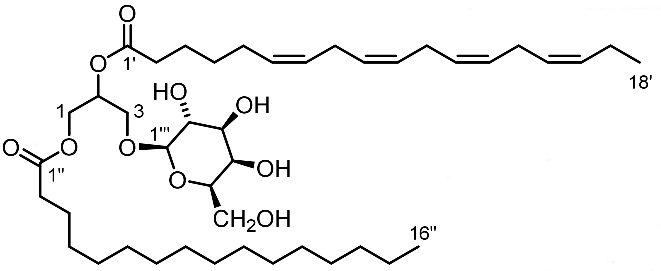

4

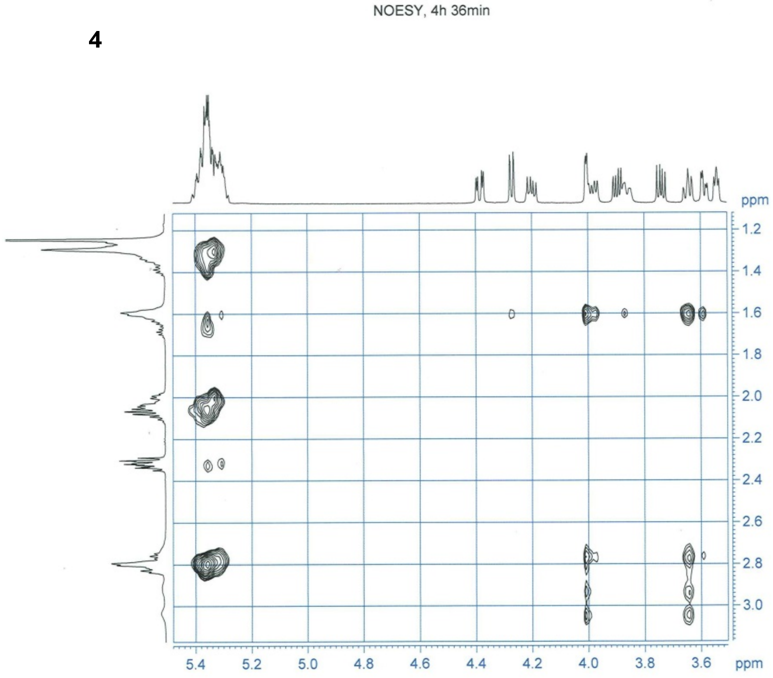

Fig. S4b Details of the NOESY spectrum of compound **4**

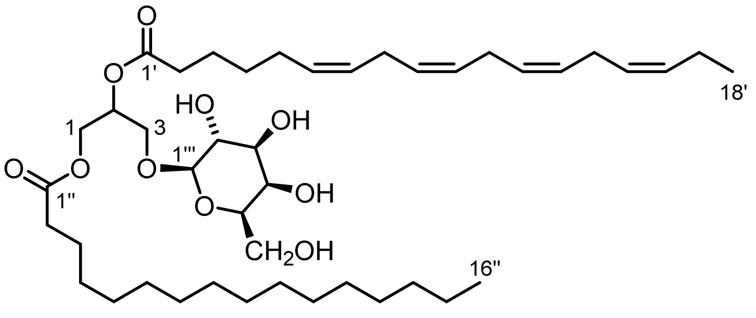

4

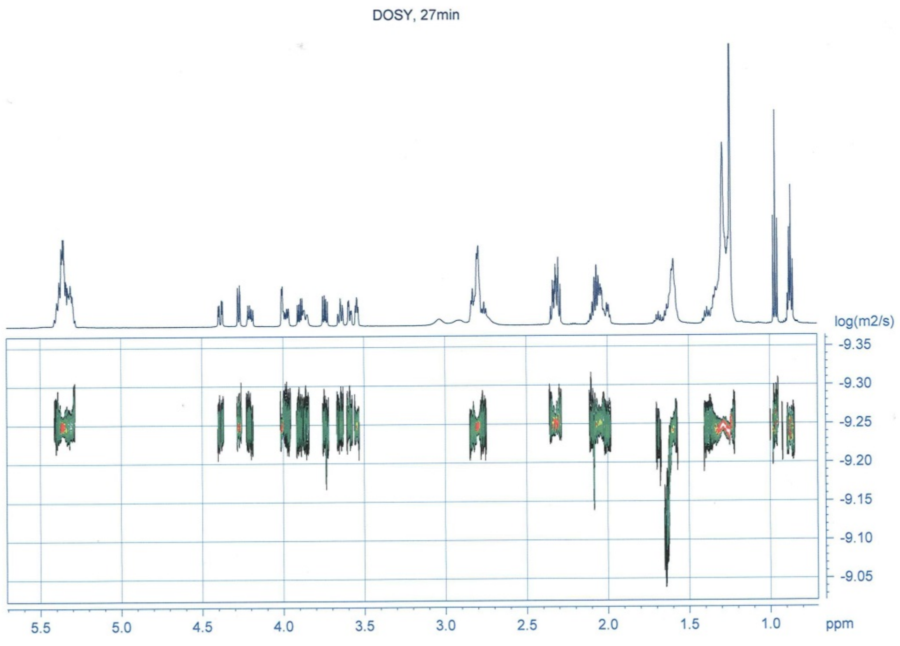

Fig. S5 DOSY spectrum of compound **4**
